# Sweet potato (*Ipomoea batatas*) and hyacinth bean (*Lablab purpureus*) in combination provide greater suppression of mile-a-minute (*Mikania micrantha*) than either crop alone

**DOI:** 10.3389/fpls.2023.1070674

**Published:** 2023-05-31

**Authors:** Shicai Shen, Gaofeng Xu, Guangzong Ma, Diyu Li, Shaosong Yang, Guimei Jin, David Roy Clements, Aidong Chen, Lina Wen, Yuchen Cui, Li Chuan, Fudou Zhang, Bo Liu

**Affiliations:** ^1^ Key Laboratory of Prevention and Control of Biological Invasions, Ministry of Agriculture and Rural Affairs of China, Agricultural Environment and Resource Research Institute, Yunnan Academy of Agricultural Sciences, Kunming, China; ^2^ Key Laboratory of Green Prevention and Control of Agricultural Transboundary Pests of Yunnan Province, Agricultural Environment and Resource Research Institute, Yunnan Academy of Agricultural Sciences, Kunming, China; ^3^ Department of Biology, Trinity Western University, Langley, BC, Canada; ^4^ School of Agriculture, Yunnan University, Kunming, China; ^5^ Agricultural Genomics Institute at Shenzhen, Chinese Academy of Agricultural Sciences, Shenzhen, China

**Keywords:** sweet potato, hyacinth bean, mile-a-minute, multispecies competition, soil nutrients

## Abstract

**Introduction:**

In natural systems, diverse plant communities tend to prevent a single species from dominating. Similarly, management of invasive alien plants may be achieved through various combinations of competing species.

**Methods:**

We used a de Wit replacement series to compare different combinations of sweet potato (*Ipomoea batatas* (L.) Lam), hyacinth bean (*Lablab purpureus* (L.) Sweet) and mile-a-minute (*Mikania micrantha* Kunth) through measures of photosynthesis, plant growth, nutrient levels in plant tissue and soil, and competitive ability.

**Results:**

Cultured alone sweet potato and hyacinth beans exhibited higher total biomass, leafstalk length, and leaf area than mile-a-minute. In mixed culture, either sweet potato or hyacinth bean or both together significantly suppressed the mile-a-minute parameters, i.e., plant height, branch, leaf, adventitious root, and biomass (P<0.05). Based on a significantly lower than 1.0 relative yield of the three plant species in mixed culture, we showed intraspecific competition to be less than interspecific competition. Calculated indices (relative yield, relative yield total, competitive balance index, and change in contribution) demonstrated a higher competitive ability and higher influence of either crop compared to mile-a-minute. The presence of sweet potato and hyacinth bean, especially with both species in combination, significantly reduced (P<0.05) mile-a-minute’s net photosynthetic rate (Pn), antioxidant enzyme activities (superoxide dismutase, peroxidase, catalase, and malondialdehyde), chlorophyll content, and nutrient content (N, P, and K). In soil with mile-a-minute in monoculture soil organic matter, total and available N, total and available K, and available P were significantly greater (P<0.05) than in soil with sweet potato grown in monoculture, but less than in soil with hyacinth bean grown in monoculture soil. Nutrient soil content was comparatively reduced for plant mixtures. Plant height, leaf, biomass, Pn, antioxidant enzyme activities, and plant and soil nutrient contents of sweet potato and hyacinth bean tended to be much greater when grown with two crops compared to in mixture with just sweet potato or hyacinth bean.

**Discussion:**

Our results suggest that the competitive abilities of both sweet potato and hyacinth bean were greater than that of mile-a-minute, and also that mile-a-minute suppression was significantly improved via a combination of the two crops compared to either sweet potato or hyacinth bean alone.

## Introduction

Plant invasions have seriously affected and threatened global biosafety and ecological security which caused serious negative impacts on native species, biodiversity, ecosystems, and environmental safety (Simberloff et al., 2013; Pyšek et al., 2020). In order to achieve ecological management of invasive alien plants, screening and competitiveness assessment of potential candidate competitor species have been conducted widely over the last 30 years. However, the inhibition effects of two or more competing species in combination on invasive alien species (Han et al., 2019) have seldom been researched for the purpose of improving crop yields in agroecosystems. Research on utilization of competition to suppress weeds has examined various ways by which a single species may be used in management, such as improvements in crop competitiveness, adjusting row spacing, crop seeding rates, or cover crop usage (Storkey et al., 2015; Sardana et al., 2017; Osipitan et al., 2018). There is growing evidence to suggest that cover crops in mixtures may be more effective in some applications than single cover crops (Storkey et al., 2015; Blesh, 2018; Baraibar et al., 2018). Likewise, multiple cropping systems such as intercropping systems show some promise in weed suppression under certain conditions (Szumigalski and Van Acker, 2005), and this potential benefit of using multiple species should likely also apply to invasive alien plant suppression. Deb (2021) highlights the need for more such research in the tropics, indicating that there are few published studies comparing crop productivity of multiple crops compared to monocultures.

Mile-a-minute (*Mikania micrantha* Kunth: Asteraceae), commonly known as plant killer in China, is a vine known for its rapid growth native to parts of Central and South America and listed as one of the 100 worst invasive species globally (Lowe et al., 2001). In Asia, mile-a-minute was first reported in Hong Kong in 1884, and now has invaded into Guangdong, Yunnan, Guangxi, Hainan, Jiangxi, and Taiwan provinces, and Hong Kong and Macao regions in China (Zhang et al., 2004; Clements et al., 2019). This plant is an aggressive weed that with vigorous biological and ecological characteristics, capable of high levels of sexual and asexual reproduction, rapid growth, high levels of adaptability, and high levels compensatory growth (Shen et al., 2013; Yang et al., 2017b; Su et al., 2021). Rapid formation of dense stands by mile-a-minute may preempt establishment by other plant species, and even kills some trees or shrubs through covering and smothering. With its ability to invade a range of habitats, mile-a-minute has left a trail of economic and ecological destruction (Zan et al., 2000; Li et al., 2007; Shen et al., 2013; Clements et al., 2019). Therefore, it is urgent for scientists and managers to explore more suitable control methods to reduce the expansion and damage caused by mile-a-minute.

Mile-a-minute usually has both prostrate growth and climbing growth patterns by which it can easily cover other plants. Mile-a-minute may produce large numbers of adventitious roots (Shen et al., 2015). These adventitious roots positively contribute to the growth of mile-a-minute, increasing plant nutrient content and soil nutrient absorption, chlorophyll content, and activities of antioxidant enzymes (Shen et al., 2021). Thus, when seeking plants to suppress the growth of mile-a-minute, the best candidates may be species that have similar niches and growth patterns to mile-a-minute. During efforts to investigate competitive control of mile-a-minute, two local important cash crops widely grown in China, were observed to be strong competitors, sweet potato [*Ipomoea batatas* (L.) Lam] (Convolvulaceae) (Shen et al., 2015), and hyacinth bean [*Lablab purpureus* (L.) Sweet] (Fabaceae) (Shen et al., 2012), and both are widely grown in China.

Native to the American tropics, sweet potato ranks seventh in importance among crops globally and fifth in developing nations (Jung et al., 2011). The normal method of propagation of sweet potato is *via* growing from roots or stem cuttings (Mohanraj and Sivasankar, 2014). Native to India, Africa, and Southeast Asia, but grown in many tropical areas, hyacinth bean serves as both a food and forage crop (Maass et al., 2010; Mthembu et al., 2018). Hyacinth bean exhibits drought resistance and high adaptability, enabling it to withstand a variety of growing conditions. It provides a potentially valuable component of sustainable cropping systems, partly due to its ability to enhance soil fertility (Maass et al., 2010). Both sweet potato and hyacinth bean are annual or perennial herbaceous vines, with sweet potato usually exhibiting a prostrate growth form and hyacinth bean exhibiting both prostrate growth and climbing growth patterns. The growth pattern and niche of sweet potato and hyacinth bean are similar to those of mile-a-minute (Shen et al., 2012; Shen et al., 2015). When grown with mile-a-minute, both sweet potato and hyacinth bean individually have been observed to suppress plant growth of mile-a-minute to some degree (Shen et al., 2012; Shen et al., 2015), but the inhibition effects of the combination of the two crops on plant growth of mile-a-minute have not been reported in the literature.

Traditional approaches to weed management have concentrated on technical solutions to reducing populations of weeds, focused narrowly on individual species. Such an approach fails to account for broader ecological interactions among diverse crops and weeds, which may offer more environmentally sustainable approaches aimed at increasing biotic resistance against weeds (Pandey and Gurr, 2019; MacLaren et al., 2020). Utilizing more than one crop competitor against weeds is a promising strategy in this ecological weed management paradigm. Using a variety of competitive plants such as cover crops or service crops represents one promising component of a more ecological approach to weed management in areas like the EU, Latin America, or tropical Asia where attempts are being made to avoid over-reliance on herbicides (Morales-Rosales and Franco-Mora, 2009; Liu et al., 2018; Deb, 2021; Tataridas et al., 2022).

Building on our previous studies (Shen et al., 2012; Shen et al., 2015), we used a de Wit replacement series approach to examine the inhibition effects of plant growth, photosynthesis and nutrient absorption of sweet potato, hyacinth bean and mile-a-minute combinations in Yunnan Province, China. Our objectives were to test whether growing the two crop species, sweet potato and hyacinth bean grown together would compete better than either crop alone and evaluate whether multiple competitive crops grown in combination could provide a sustainable invasive plant management system.

## Materials and methods

### Plant materials

The source of mile-a-minute plants in our experiments was a Mangshi City population in Dehong Prefecture, Yunnan Province, China. Likewise sweet potato for our experiments was collected and propagated in Mangshi City. Both sweet potato and hyacinth bean are widely grown throughout the tropical and subtropical regions of China. Seeds of hyacinth bean were collected from local populations in September in 2019, dried for two months at room temperature, and then stored at -4°C. Although the two crops, sweet potato and hyacinth bean, and mile-a-minute have different geographic origins, conditions in Dehong Prefecture with average monthly rainfall of 1595 mm and average monthly temperatures of 18.9°C (Shen et al., 2015), are highly favorable for the growth of all three species.

### Experiment design and data collection

A de Wit replacement series (de Wit., 1960) was set up on farmland in Zhefang Town, Mangshi City, over the June to October 2020 growing season. The plant materials for sweet potato and mile-a-minute were collected and treated using similar methods to those used in a previous study (Shen et al., 2015). This involved deriving one-node sections from similar-sized plants of both species (sweet potato and mile-a-minute). On June 10^th^, 5–6 cm one-node segments weighing 2.0–2.5 g (fresh weight) were extracted from central stem portions of sweet potato and mile-a-minute plants propagated in a greenhouse. The extracted segments were immersed in pure water and grown for 7 days. On June 17^th^, the sprouts derived from cuttings of sweet potato and mile-a-minute plants with similar height were selected and transplanted, and the seeds of hyacinth bean were sown into 16 m^2^ plots (4 m × 4 m). Six ratios of mile-a-minute grown in combination with one or two crop species (sweet potato versus mile-a-minute, hyacinth bean versus mile-a-minute, and sweet potato versus mile-a-minute versus hyacinth bean) were set up to add up to 320 plants per treatment (2:0/320:0 sweet potato plants, 1:1/160:160 sweet potato versus mile-a-minute plants, 1:1/160:160 hyacinth bean versus mile-a-minute plants, 0.5:1:0.5/80:160:80 sweet potato versus mile-a-minute versus hyacinth bean plants, 0:2/0:320 mile-a-minute plants, 0:2/0:320 hyacinth bean plants) with a planting density kept constant at 20 plants m^−2^ (0.25 m × 0.20 m plot space). The ratios were determined based on our experience from previous experiments and field observations of sweet potato and mile-a-minute. Plots were arranged in a complete randomized design with 16 replicates per ratio. Within each plot, plants were evenly distributed. A 1.5 m border was constructed between plots and each plot was fenced with 0.35 m high glass panels to prevent the plants from climbing beyond the plots. Plots were hand-weeded and were not fertilized with synthetic fertilizers.

During the experiment, a random harvest of twenty-five plants of each species was taken from the interiors of the plots in July, August, and September. Four plots with same ratio were randomly selected and destroyed each time. Measurements were taken for total shoot length, main stem length, branch number, leafstalk length, leaf area, and aboveground fresh biomass. From mid-July to mid-October, net photosynthetic rate (Pn) measurements were recorded for sweet potato, mile-a-minute, and hyacinth bean. The measurements were taken between 8:00 am and 11:30 am using a Portable Photosynthesis System (LI-COR Biosciences LI6400XT, Lincoln), with a 6400-02 or -02B LED source and 1000 µmol m^-2^ s^-1^ photosynthetically active radiation. Ambient air values of 375 ± 3 ppm CO_2_, 25 ± 1°C and 65% ± 10% RH were maintained during sampling, by matching these values in the chamber. Five to six randomly selected individual plants of each species were scanned in order to choose a random leaf from which to take measurements.

The experiment went for 125 days and was terminated on 20 October 2020. At that point, measurements of total shoot length, main stem length, branch number, leafstalk length, leaf area, and aboveground fresh biomass of twenty-five plants of each species were made for the last four plots belonging to each ratio. Leaf area index was determined *via* a leaf-area meter (Li-3000A; Li-Cor Corp.). Enzyme extracts and assays were also obtained for 5–6 g leaf samples from the three species immediately frozen in liquid nitrogen. Sample plant leaves were also collected for plant nutrient analysis, and subject to drying for 96 h at 40 ± 5°C. Antioxidant enzyme activities for superoxide dismutase (SOD), catalase (CAT), peroxidase (POD), and malondialdehyde (MDA) of plant leaves were measured and analyzed at the Agricultural Environment and Resources Research Institute of Yunnan Academy of Agricultural Sciences (Zhang et al., 2016; Cheng et al., 2018).

To control for soil nutrient changes, 25 soil samples (0–10 cm) were taken randomly at the site prior to the experiment and combined as a composite sample. At the time of the last harvest, fifty soil samples were taken randomly from each plot and then mixed to form one composite sample. Soils were ground and sifted using a 2 mm sieve and air-dried at room temperature. Soil nutrient analysis was completed at the Soil Analysis and Detection Center of the Agricultural Environment and Resource Research Institute, Yunnan Academy of Agricultural Sciences, China. In terms of plant nutrient content, we recorded N, P, and K, and our soil nutrient analysis included pH, soil organic matter, total N, total P, total K, available N, available P, and available K.

### Data analyses

The final biomass data for each species and treatment was used to calculate the relative yield (RY) (de Wit, 1960) per plant, the relative yield total (RYT) (Fowler, 1982), and competitive balance index (CB) (Wilson, 1988). The final biomass for treatments containing all three species was also used to assess change in contribution (CC) (Williams and McCarthy, 2001). Analysis of variance (one-way ANOVA using IBM SPSS 23.0 software (Armonk)) was conducted on plant morphological variables (stem length, branch, number of nodes with adventitious roots, and biomass), plant nutrient content (N, P and K), soil parameters (pH, soil organic matter, total N, total P, total K, available N, available P, and available K), and physiological variables (Pn, antioxidant enzymes, and chlorophyll). Tukey’s HSD, *Post Hoc* Multiple Comparison, and Homogeneity of Variance tests were used to examine differences among treatments.

## Results

### Plant growth

**Figure 1:**
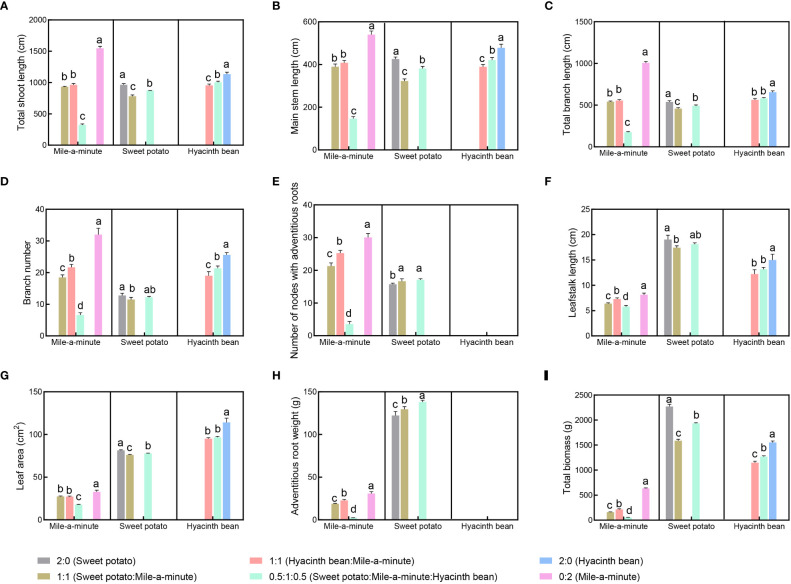
Plant growth comparison of three species looking at total shoot length **(A)**, main stem length **(B)**, total branch length **(C)**, branch number **(D)**, number of nodes with adventitious roots **(E)**, leafstalk length **(F)**, leaf area **(G)**, adventitious root biomass **(H)**, and total biomass **(I)** of sweet potato, hyacinth bean and mile-a-minute competition under mono and mixed culture conditions. Different letters signify significant differencesat P<0.05.

Comparing growth of the three species in monoculture, main stem length and branch length of mile-a-minute were higher than sweet potato and hyacinth bean main stem length and branch length (**Figure 1**). However, in mixed culture there was significant suppression of mile-a-minute total shoot length (main stem + branch length), main stem length and branch length (P < 0.05). These growth indices were reduced in the presence of just one competing species but reduced much more in the presence of both competing species, by more than twice as much for main stem length and nearly twice as much for branch length. Reduction of the main stem length and branch length of mile-a-minute amounted to 27.8% and 46.2%, respectively, for a 1:1 ratio of sweet potato to mile-a-minute, 24.4% and 44.7%, respectively, for 1:1 ratio of hyacinth bean to mile-a-minute, and 72.8% and 82.5%, respectively, for a 0.5:1:0.5 ratio of sweet potato versus mile-a-minute versus hyacinth bean.

Comparing branching parameters for the monocultures, the branch number and number of nodes with adventitious roots were greater for mile-a-minute than sweet potato (**Figure 1**). Note that hyacinth bean does not produce adventitious roots. In mixed culture, suppression of the branch number and number of nodes with adventitious roots was evidently suppressed for mile-a-minute (P<0.05), with mile-a-minute being inhibited more than sweet potato. Mile-a-minute branch number and number of nodes possessing adventitious roots was reduced by 42.2% and 29.1%, respectively, for a 1:1 ratio of sweet potato to mile-a-minute, 32.2% and 15.9%, respectively for a 1:1 ratio of hyacinth bean to mile-a-minute, and 79.4% and 88.2%, respectively for a 0.5:1:0.5 ratio of sweet potato versus mile-a-minute versus hyacinth bean. As seen in main stem and branch length, the combination of the two competitors had a markedly greater impact on mile-a-minute branch number and adventitious roots (**Figure 1**).

In all treatments, the leafstalk length and leaf area of sweet potato and hyacinth bean were markedly higher than those of mile-a-minute (**Figure 1**). Comparing leaf parameters in monocultures of the three species, the mean leafstalk length and leaf area were 19.01 cm and 81.32 cm^2^ for sweet potato, 14.98 cm and 114.10 cm^2^ for hyacinth bean and only 8.13 cm and 32.76 cm^2^ for mile-a-minute. In mixed culture, the leafstalk length and leaf area of mile-a-minute progressively declined even further with the combination both sweet potato and hyacinth bean having a noticeably greater effect than either species alone, and sweet potato and hyacinth bean were significantly less suppressed in mixed cultures (**Figure 1**).

Sweet potato exhibited much greater adventitious root biomass than mile-a-minute across all treatments (**Figure 1**). In mixed culture, significant suppression of the adventitious root biomass of mile-a-minute was observed (P<0.05), whereas the adventitious root biomass of sweet potato markedly increased. The total biomass of mile-a-minute was much less than that of sweet potato and hyacinth bean in all treatments. The significant reduction in mile-a-minute biomass was accompanied by higher inhibition rates in mixed culture. The adventitious root biomass and total biomass of mile-a-minute declined by 92.4% and 91.9%, respectively, at the 0.5:1:0.5 sweet potato:mile-a-minute:hyacinth bean ratio in mixed culture (**Figure 1**).

### Competitive interactions

**Figure 2:**
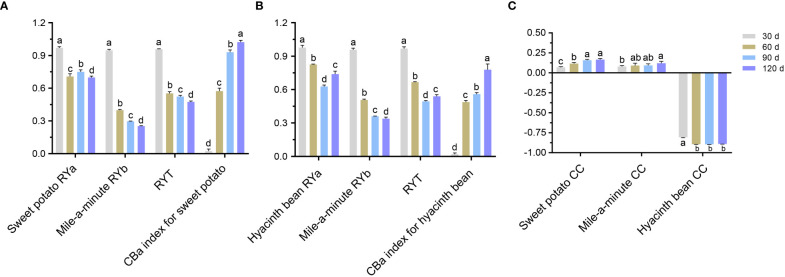
Relative yield (RY), relative yield total (RYT), competitive balance (CB) index, change in contribution (CC) of sweet potato, hyacinth bean and mile-a-minute under mono and mixed culture conditions. 1:1 (Sweet potato:Mile-a-minute) **(A)**, 1:1 (Hyacinth bean:Mile-a-minute) **(B)** and 0.5:1:0.5 (Sweet potato:Mile-a-minute:Hyacinth bean) **(C)**. Different letters signify significant differences at P<0.05.

Competition between mile-a-minute and each of the two crop species was uneven and favored sweet potato and hyacinth bean as indicated by relative yield (RY) values (**Figure 2**). Our results also showed that intraspecific competition was less than interspecific competition between mile-a-minute and the crop species. This was seen in the RY values significantly less (P<0.05) than 1.0 in mixed culture. Likewise the relative yield total (RYT) for each species was less than 1.0 in mixed culture (P<0.05), signaling that significant competition was taking place between both crops and mile-a-minute. The competitive balance (CB) index for the two crop species was greater than zero, and the CB index of sweet potato was higher than that of hyacinth bean in mixed culture showing sweet potato was very competitive. The change in contribution (CC) of the two crops were significantly higher than that of mile-a-minute indicated that sweet potato and hyacinth bean had greater influence (as measured by change in contribution CC) on the community when all three plant species were grown together. The RY, CB and CC of sweet potato and hyacinth bean were significantly increased, the CB and CC of sweet potato were higher than those of hyacinth bean within same growth period, and the RY and CC of mile-a-minute were reduced with increasing densities of the two crops from July to October. Together these results demonstrated that the two crops grown in concert outranked the competitive ability of mile-a-minute, and sweet potato had greater competitive ability than hyacinth bean.

### Physiological parameters

The Pn of sweet potato, mile-a-minute and hyacinth bean showed incremental increases from July to October regardless of treatment (**Table 1**). Comparing plants grown in monoculture, Pn values for sweet potato and hyacinth bean from July to August were higher than mile-a-minute Pn values, but less than mile-a-minute in September and October. Looking at Pn values in July, sweet potato and hyacinth bean exhibited significantly higher values than mile-a-minute (P<0.05). By August and in the following months, Pn values for mile-a-minute, sweet potato and hyacinth bean were inhibited significantly (P < 0.05) in the presence of competitors, but the Pn of sweet potato and hyacinth bean in the three-species treatment was much greater than when either crop was grown individually with mile-a-minute (**Table 1**).

**Table 1:** Net photosynthetic rate (Pn) of sweet potato, hyacinth bean and mile-a-minute competition under mono and mixed culture conditions.

Variables		Ratios					
		2:0 (Sweet potato)	1:1 (Sweet potato: Mile-a-minute)	1:1 (Hyacinth bean: Mile-a-minute)	0.5:1:0.5 (Sweet potato:Mile-a-minute: Hyacinth bean)	2:0 (Hyacinth bean)	0:2 (Mile-a-minute)
July	Mile-a-minute Pn (µmol CO_2_ m^-2^ s^-1^)	–	9.01 ± 0.10a	9.07 ± 0.22a	8.41 ± 0.14b	–	9.00 ± 0.09a
	Sweet potato Pn (µmol CO_2_ m^-2^ s^-1^)	9.36 ± 0.11a	9.33 ± 0.11a	–	9.51 ± 0.11a	–	–
	Hyacinth bean Pn (µmol CO_2_ m^-2^ s^-1^)	–	–	10.36 ± 0.21a	10.40 ± 0.22a	10.32 ± 0.16a	–
August	Mile-a-minute Pn (µmol CO_2_ m^-2^ s^-1^)	–	11.12 ± 0.08c	11.36 ± 0.13b	9.04 ± 0.08d	–	11.55 ± 0.08a
	Sweet potato Pn (µmol CO_2_ m^-2^ s^-1^)	13.28 ± 0.28a	12.91 ± 0.13b	–	13.12 ± 0.15ab	–	–
	Hyacinth bean Pn (µmol CO_2_ m^-2^ s^-1^)	–	–	11.78 ± 0.33b	12.54 ± 0.19a	12.32 ± 0.20a	–
September	Mile-a-minute Pn (µmol CO_2_ m^-2^ s^-1^)	–	12.97 ± 0.21c	13.40 ± 0.10b	9.50 ± 0.14d	–	15.03 ± 0.26a
	Sweet potato Pn (µmol CO_2_ m^-2^ s^-1^)	14.31 ± 0.21b	13.08 ± 0.12c	–	14.91 ± 0.10a	–	–
	Hyacinth bean Pn (µmol CO_2_ m^-2^ s^-1^)	–	–	13.13 ± 0.19b	13.49 ± 0.12a	13.34 ± 0.21ab	–
October	Mile-a-minute Pn (µmol CO_2_ m^-2^ s^-1^)	–	13.39 ± 0.13b	13.68 ± 0.18b	10.17 ± 0.09c	–	16.27 ± 0.38a
	Sweet potato Pn (µmol CO_2_ m^-2^ s^-1^)	15.38 ± 0.18a	14.52 ± 0.25c	–	15.52 ± 0.26b	–	–
	Hyacinth bean Pn (µmol CO_2_ m^-2^ s^-1^)	–	–	13.47 ± 0.29c	14.45 ± 0.18b	14.92 ± 0.10a	–

Data are expressed as mean ± standard deviation. Different letters within the same row signify significant differences at P < 0.05.

When comparing plants grown in monoculture, mile-a-minute exhibited greater POD and SOD antioxidant enzyme activities than those seen for sweet potato and hyacinth bean, whereas the opposite relationship was seen for CAT and MDA antioxidant activities (**Table 2**). In mixed culture, the SOD and CAT activities of all three species were significantly suppressed, and the inhibition rates were ranked in order from the greatest inhibition for all 3 species together, to moderate inhibition for sweet potato in combination with mile-a-minute, followed by the least inhibit for hyacinth bean and mile-a-minute. For both CAT and MDA, the highest activity levels occurred when all three species were grown together (**Table 2**).

**Table 2:** Antioxidant enzyme and chlorophyll properties of sweet potato, hyacinth bean and mile-a-minute competition under mono and mixed culture conditions.

Variables		Ratios					
		2:0 (Sweet potato)	1:1 ( Sweet potato:Mile-a-minute)	1:1 (Hyacinth bean: Mile-a-minute)	0.5:1:0.5 (Sweet potato: Mile-a-minute:Hyacinth bean)	2:0 (Hyacinth bean)	0:2 (Mile-a-minute)
SOD (U·g^-1^)	Mile-a-minute	–	90.07 ± 3.48c	112.77 ± 6.85b	52.55 ± 3.24d	–	145.46 ± 5.85a
	Hyacinth bean	–	–	75.76 ± 2.66b	79.92 ± 2.59b	93.26 ± 5.76a	–
	Sweet potato	88.34 ± 3.04a	82.34 ± 1.55b	–	84.87 ± 1.87ab	–	–
CAT (U·g^-1^)	Mile-a-minute	–	13.05 ± 0.29d	11.30 ± 0.23b	10.41 ± 0.22c	–	9.63 ± 0.19d
	Hyacinth bean	–	–	43.56 ± 0.50b	45.08 ± 0.42a	41.80 ± 0.91c	–
	Sweet potato	21.47 ± 0.34c	22.94 ± 0.22b	–	25.11 ± 0.30a	–	–
POD (U·g^-1^)	Mile-a-minute	–	46.21 ± 0.64d	68.65 ± 0.39b	53.74 ± 0.41c	–	75.55 ± 0.66a
	Hyacinth bean	–	–	7.55 ± 0.14c	9.07 ± 0.13b	11.48 ± 0.58a	–
	Sweet potato	31.48 ± 0.47a	24.66 ± 0.59c	–	28.56 ± 0.43b	–	–
MDA (nmol·g^-1^)	Mile-a-minute	–	82.88 ± 1.90a	25.99 ± 0.45c	47.45 ± 1.71b	–	22.90 ± 0.85d
	Hyacinth bean	–	–	47.79 ± 1.37b	92.99 ± 2.5a	42.42 ± 1.87c	–
	Sweet potato	61.87 ± 1.26c	89.05 ± 2.24b	–	95.73 ± 1.17a	–	–
Chlorophyl (nmol·g^-1^)	Mile-a-minute	–	2.05 ± 0.06c	2.24 ± 0.04b	1.59 ± 0.04d	–	2.72 ± 0.05a
	Hyacinth bean	–	–	3.55 ± 0.07b	3.63 ± 0.03b	4.47 ± 0.14a	–
	Sweet potato	4.72 ± 0.05a	3.93 ± 0.03c	–	4.23 ± 0.09b	–	–

Data are expressed as mean ± standard deviation. Different letters within same row signify significant differences at P<0.05.

SOD, superoxide dismutase; CAT, catalase; POD, peroxidase; MDA, malondialdehyde.

Chlorophyll content of sweet potato and hyacinth bean was significantly greater than that of mile-a-minute in all treatments (**Table 2**). The chlorophyll content of mile-a-minute was significantly suppressed (P < 0.05) in competition with crops, with both crops in combination creating the greatest reduction in chlorophyll content in mile-a-minute.

### Plant and soil nutrient effects

Plant N and P content of either sweet potato or hyacinth bean was usually greater than for mile-a-minute, but the K contents of sweet potato and hyacinth bean were significantly less those of mile-a-minute regardless of treatment (**Table 3**). In mixed culture, plant N, P and K contents of all three species were significantly reduced when grown with other species, with the greatest inhibition rates seen for the combination of all three species with the treatment indicating that the presence of hyacinth bean improved the soil nutrient absorption ability of sweet potato.

**Table 3:** Plant nutrient contents (i.e., N, P, and K) of sweet potato, hyacinth bean and mile-a-minute competition under mono and mixed culture conditions.

Variables		Ratios					
		2:0 (Sweet potato)	1:1 (Sweet potato:Mile-a-minute)	1:1 (Hyacinth bean:Mile-a-minute)	0.5:1:0.5 (Sweet potato:Mile-a-minute:Hyacinth bean)	2:0 (Hyacinth bean)	0:2 (Mile-a-minute)
N content (mg/kg)	Mile-a-minute	–	21.02 ± 0.67c	23.30 ± 0.34b	17.03 ± 0.26d	–	25.09 ± 0.25a
	Hyacinth bean	–	–	33.53 ± 0.53c	38.11 ± 0.29b	44.10 ± 0.31a	–
	Sweet potato	35.20 ± 0.62a	31.37 ± 0.49c	–	32.93 ± 0.22b	–	–
P content (mg/kg)	Mile-a-minute	–	1.97 ± 0.10c	2.17 ± 0.06b	1.36 ± 0.06d	–	2.93 ± 0.07a
	Hyacinth bean	–	–	2.92 ± 0.02c	3.15 ± 0.08b	3.61 ± 0.12a	–
	Sweet potato	2.87 ± 0.06a	2.39 ± 0.03c	–	2.53 ± 0.05b	–	–
K content (mg/kg)	Mile-a-minute	–	22.57 ± 0.81c	24.25 ± 0.33b	16.18 ± 0.45d	–	28.19 ± 0.44a
	Hyacinth bean	–	–	13.69 ± 0.11c	14.41 ± 0.24b	16.55 ± 0.26a	–
	Sweet potato	15.19 ± 0.39a	11.37 ± 0.25c	–	14.13 ± 0.17b	–	–

Data are expressed as mean ± standard deviation. Different letters within same row signify significant differences at P < 0.05.

**Figure 3:**
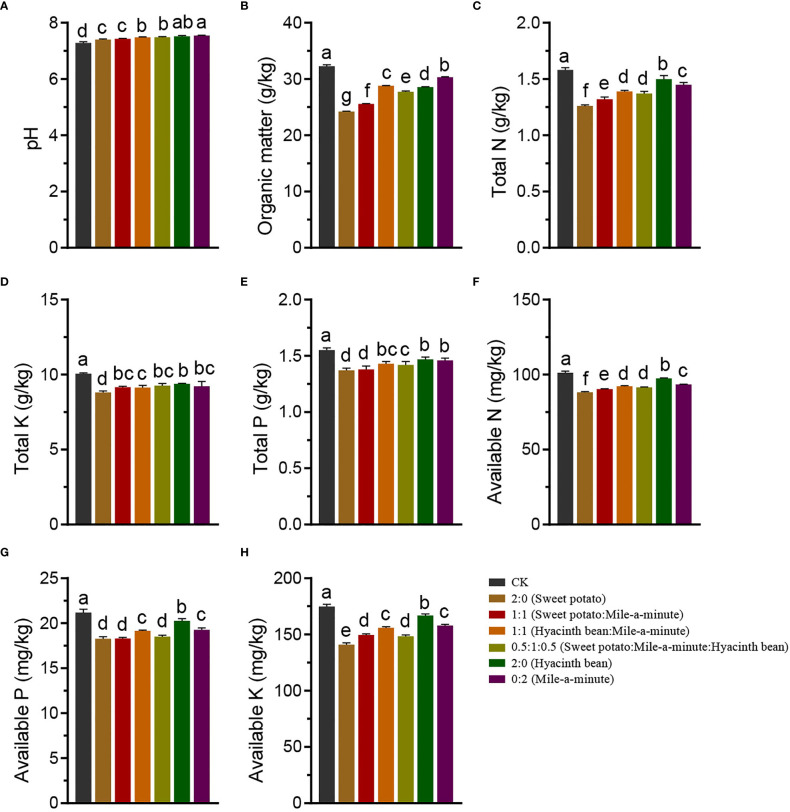
Soil properties **looking at** pH **(A)**, organic matter **(B)**, total N **(C)**, total K **(D)**, total P **(E)**, available N **(F)**, available P **(G)**, and available K **(H)** of sweet potato, hyacinth bean and mile-a-minute competition under mono and mixed culture conditions. Different letters signify significant differences at P < 0.05.

The pH of initial soil (CK) was slightly lower than the pH in the various treatments at harvest time. Comparing soil contents for plants grown in monoculture, we showed total N, total P, total K, available N, available P, and available K content were all significantly higher (P<0.05) for hyacinth bean than for either sweet potato or mile-a-minute. Mixed culture soils with hyacinth bean generally exhibited greater nutrient content values than soils without hyacinth bean (**Figure 3**), demonstrating the tendency of hyacinth bean to improve soil characteristics even in competitive situations.

## Discussion

The current study demonstrated that sweet potato and hyacinth bean combinations could achieve a high level of suppression of mile-a-minute. The combination of both crop species consistently exhibited greater suppression of mile-a-minute than either species alone. In comparison to mile-a-minute, sweet potato, and hyacinth bean each possessed superior attributes in terms of plant leaf area, biomass production, photosynthesis, and soil nutrient absorption ability, and displayed greater competitive ability than mile-a-minute when the plants were grown together. Morphological characteristics (e.g., leaf shape) and biomass are generally regarded as the best measures of competitive ability (Keddy et al., 2002; Jiang et al., 2008). These characteristics can be incorporated into indices such as relative yield (RY) or competitive balance (CB) to help demonstrate their competitive impact on neighboring plants (Williams and McCarthy, 2001). Previous studies showed that sweet potato had stronger competitive ability than a variety of highly invasive alien plants including mile-a-minute, *Ageratum conyzoides*, *Ageratina adenophora*, *Bidens pilosa*, and *Galinsoga parviflora* (Shen et al., 2015; Shen et al., 2019a; Shen et al., 2019b) and hyacinth bean was very competitive towards *Parthenium hysterophorus* (Ojija and Ngimba, 2021). In the current study we likewise found that sweet potato and hyacinth bean both competed strongly with mile-a-minute, and that it was important to assess the competitive relationship through the whole growing season. The competitiveness of sweet potato and hyacinth bean gradually increased from July to October, although hyacinth bean was generally less competitive than sweet potato. However, the fact that hyacinth bean in combination with sweet potato caused much greater suppression of mile-a-minute than sweet potato alone revealed a synergism between the two crops when in combination. This may have been partly due to differentiation of the two crops in vertical space, with sweet potato growing underneath mile-a-minute and hyacinth bean overtopping mile-a-minute. The two crops sandwiching the weed between them would help to reduce its normally expansive growth form.

The three plant species sweet potato, hyacinth bean and mile-a-minute are all classed as vines therefore sharing similar niches and possess many morphological similarities. When grown with some erect invasive plant species, the intraspecific competition of sweet potato was usually greater than its interspecific competition (Shen et al., 2019a; Shen et al., 2019b). However, our study found that the intraspecific competition between two crops and mile-a-minute was less than their interspecific competition, demonstrating that the plant growth of sweet potato and hyacinth bean was also inhibited by mile-a-minute in mixed culture. Sweet potato is mainly propagated by clonal means and the seedlings grow quickly and can easily cover the available soil surface. Hyacinth bean is propagated by seeds, with high germination rate and rapid seedling growth (Maass, 2006). Moreover, this bean crop is drought resistant, highly adaptable, and used for soil conservation, and soil fertility improvement (Maass et al., 2010). In South Africa, Mthembu et al. (2018) found that intercropping with hyacinth bean in maize created a more sustainable system, potentially reducing the need for herbicide applications. Mile-a-minute has a high reproductive output, both asexually and sexually, but its seedling grows slowly by comparison to many other species (Shen et al., 2016). Desirable qualities of crop plants utilized to outcompete invasive plants include ease of propagation, high economic value, and ability to fill in the canopy rapidly (Yang et al., 2017a). Sweet potato and hyacinth bean exhibit rapid growth, vigorous root systems, ability to form a canopy rapidly, branching capacity, efficient biomass production, and both species are widely used as food and cash crops, fodder, and medicinal plants (Maass et al., 2010; Shen et al., 2012; Shen et al., 2015; Mthembu et al., 2018). The current study showed that both sweet potato and hyacinth bean are suitable for being planted to compete with mile-a-minute.

The general success of mile-a-minute in cropping situations lies in its ability to occupy the invaded habitat and cover other plants with its long and thin main stem and branch length and large number of branches, all contributing to its rapid growth and population expansion. However, in our study, either sweet potato or hyacinth bean could suppress the main stem length, branch length, and branch number of mile-a-minute were significantly suppressed, and to a much greater extent with both crop species present. Plants like mile-a-minute use their branching ability to pre-empt resources, making them no longer available for would-be competitors (Jiang et al., 2008). Comparing growth in monocultures, the branch number of mile-a-minute exceeded that of either sweet potato or hyacinth bean, but its branch number was severely suppressed in mixed culture, while the sweet potato branch number simultaneously increased. Thus, as in the case of other multiple cropping systems being tested in the tropics (Morales-Rosales and Franco-Mora, 2009; Liu et al., 2018; Deb, 2021), incorporating both sweet potato and hyacinth bean shows greater promise than either species planted as a competitive crop alone. Increasing biotic resistance in agroecosystems is often said to involve diversifying plant functional types (MacLaren et al., 2020), while in our specific system it is a matter of beating the invasive species at its own game, using two other vines as crops. Over the long term, a system relying on such direct competition might be affected by the potential for mile-a-minute to evolve new attributes (Yang et al., 2017b; Su et al., 2021) to compete better with sweet potato and hyacinth bean, so it would be important to employ other integrated weed management techniques in addition to plant-plant competition.

Plant leaves are key to measuring the efficiency by which plants utilize solar energy (Baldwin and Schmelz, 1994), and we detected major differences in leaf area index depending on plant species composition. Greater specific leaf area, Pn and chlorophyll represent greater investments in carbon fixation (Lambers and Poorter, 1992). In our study this greater investment by sweet potato or hyacinth bean than in mile-a-minute was seen in significantly greater leafstalk length and leaf area of sweet potato and hyacinth bean. In monoculture treatments, leafstalk length and leaf area of mile-a-minute comprised only 43% and 54% of those of sweet potato, and 37% and 26% of those of hyacinth bean, respectively. Average mile-a-minute leafstalk length and leaf area were reduced in mixed culture, with the greatest reduction seen in the presence of both crops when the leaf stalk length and leaf area of mile-a-minute were reduced by 29% and 46%, of their values in monoculture, respectively. The ranking of net photosynthetic rates (Pn) varied over the season. In monoculture conditions, mile-a-minute exhibited higher Pn than the two crops from September to October even though it lagged behind the crops earlier in the season. Yet when grown with the crops, its Pn was suppressed in August and subsequent months. Sweet potato competition has likewise been shown to exert its competitive ability through leaf area and Pn in other studies (Shen et al., 2019a; Shen et al., 2019b). In the present study, leaf area, Pn and chlorophyll content of sweet potato and hyacinth bean when grown together than individually in competition with mile-a-minute, indicating synergistic effects were at play, and it was clear that over time as the growing period progressed, the differences in photosynthetic ability and output became more pronounced.

Antioxidant enzymes are an important physiological strategy for plants in response to environmental stresses, and sweet potato and hyacinth bean have been shown to have greater ability to increase antioxidant enzyme production when in competition with invasive plants (Shen et al., 2019b). Antioxidant enzyme activity levels reflect the ability of plants to resist disease and insect attacks as well as other stresses on plant physiology such as adverse environmental conditions (Zhang et al., 2009). Zhang and Wen (2008) demonstrated that infestation of mile-a-minute by the insect *Bemisia tabaci* resulted in significantly decreased SOD and CAT activities, and consequently harmful active oxygen species (AOS) were more difficult to eliminate (Zhang and Wen, 2008). Antioxidant enzyme activities (SOD, CAT, POD, and MDA) were shown to increase for mile-a-minute when plants bore more nodes with adventitious roots (Shen et al., 2021). Competitive relationships have been shown to influence SOD, POD and CAT activities, such in the case of competition between sweet potato and the invasive plant *Ageratina adenophora* (Shen et al., 2019a). In that study, SOD, POD and CAT activity was tied to portions of the respective species in mixture, with the species present in higher portion exhibiting higher activity. This pattern was also seen for SOD, POD and CAT activity of invasive plant species *A. conyzoides*, *B. pilosa*, and *G. parviflora* in competition with sweet potato (Shen et al., 2019b). Our present study showed that in monocultures, POD and SOD activity levels were greater in mile-a-minute than for either sweet potato or hyacinth bean, whereas CAT and MDA activity levels for mile-a-minute outranked activity in the two crops. In mixed culture, mile-a-minute SOD and POD activity was significantly suppressed, with inhibition rates ranked in order from the combination of both crops growing with mile-a-minute (greatest), versus sweet potato growing with mile-a-minute (moderate) versus hyacinth bean growing with mile-a-minute (least). MDA activity was highest for both crops growing in competition with mile-a-minute, particularly sweet potato that exhibited 44% more MDA activity with mile-a-minute only, and 55% more when grown with both other species. Clearly the combination of both crops could improve the antioxidant enzyme activities and MDA of sweet potato and hyacinth bean to resist the negative impact by mile-a-minute.

Plant N, P, K contents are closely related to their absorption capacity and available soil nutrients. Higher plant nutrient content and lower soil nutrient content demonstrates stronger absorption capacity of plants. Both sweet potato and mile-a-minute produce large numbers of adventitious roots from the main stem and branch nodes as stimulated by contact with the soil surface (Shen et al., 2015). The adventitious roots of mile-a-minute may positively contribute to its plant nutrient contents and soil nutrient absorption ability (Shen et al., 2021). Sweet potato growth was particularly sensitive to fertilization level whereas under various fertility levels the density of competitors was more important than fertilization for mile-a-minute (Shen et al., 2020). Fixation of atmospheric nitrogen by hyacinth bean may enrich soil fertility (Kaldate et al, 2021). A previous study showed that soil nutrients were depleted during the growth of sweet potato and mile-a-minute, and with the two plants grown in mixture, sweet potato was able to consume more nutrients (Shen et al., 2015). The present study found that plant N and P contents of sweet potato and hyacinth bean outranked those of mile-a-minute. In mixed culture, plant N, P and K contents for each of the three species were significantly reduced with increasing numbers of competitors, with the greatest inhibition seen for all three species growing together, followed by sweet potato growing with mile-a-minute, and lastly hyacinth bean growing with mile-a-minute. The N, P, and K contents of sweet potato were greater when growing with both other species than in monoculture indicating that the soil nutrient absorption ability of sweet potato was improved *via* hyacinth bean. Moreover, the total N content, total P content, total K content, available N content, available P content, and available K content of hyacinth bean soil were significantly higher in monoculture than those of sweet potato and mile-a-minute. In mixed culture including hyacinth bean, soil nutrient levels were usually greater than in soils without hyacinth bean, showing that the soil characteristics were improved *via* hyacinth bean. Monitoring changes to both soil and plant nutrient content over the growing season, enabled us to observe that when hyacinth bean was present, nutrient uptake by sweet potato significantly increased. Sitienei et al. (2017) demonstrated that a maize/hyacinth bean intercrop increased available P and K content of soils, and greater maize yields were achieved compared to maize alone (Sitienei et al., 2017). The total N content of soils in maize monoculture and maize/hyacinth bean intercrop was not much different because no fertilizer was used (Sitienei et al., 2017). Furthermore, hyacinth bean cropping systems have been observed to exhibit higher N release rates *via* biological fixing of N and decomposition (Ayoub, 1986).

## Conclusion

The present study demonstrated that the combination of sweet potato and hyacinth bean could achieve greater suppression of mile-a-minute than either crop species alone. Many different relevant parameters of mile-a-minute were reduced when grown with sweet potato and hyacinth bean, such as stem length, branch number, adventitious root, biomass, Pn, antioxidant enzyme activities, chlorophyll content, plant nutrient content, and soil nutrient absorption ability. Meanwhile many sweet potato and hyacinth bean parameters were markedly greater when both crops were grown in combination with mile-a-minute than the parameters for each crop species acting as a single competitor with mile-a-minute. Moreover, both the soil nutrient acquisition and competitive ability of sweet potato saw improvement when grown with hyacinth bean as a co-competitor. Thus, two or more competitive species could provide a strategic tool for suppressing mile-a-minute growth, and optimal plant species and density ratios could be developed from our study to produce high sweet potato yields while simultaneously managing invasive plants. The application of our experimental trial results requires further testing, ideally scaling up our small-scale results to the farm level. Further research is needed to provide additional details of the morphological or physiological impacts of sweet potato and hyacinth bean combinations or other multispecies combinations on mile-a-minute and other invasive alien plants. Over time, some mile-a-minute genotypes may evolve to better compete with these two species, so it is important to apply a diverse set of management techniques to suppress this invasive species. Use of several crops in combination promises to improve overall biotic resistance of agroecosystems to reduce the impact of invasive plants. Efforts should also be made to understand multispecies competitive mechanisms more fully to help demonstrate their potential integration into existing cropping systems.

## Data availability statement

The original contributions presented in the study are included in the article/supplementary material. Further inquiries can be directed to the corresponding author.

## Author contributions

SS conceived and designed the experiments, performed the experiments, analyzed the data, and wrote the draft of the manuscript. GM performed the experiments and analyzed the data. GX, GM, DL, SY, GJ, AC, LW, YC, LC, and BL performed the experiments. DC analyzed the data and drafted the manuscript. FZ conceived and designed the experiments and performed the experiments. All authors read and approved the final manuscript. All authors contributed to the article.
